# European Federation of Periodontology Survey of Postgraduate and Specialist Training in Europe in 2020

**DOI:** 10.1111/eje.12711

**Published:** 2021-08-21

**Authors:** Kenneth A. Eaton, Nicola X. West, Nairn H. F. Wilson, Mariano Sanz

**Affiliations:** ^1^ Eastman Dental Institute University College London and Honorary Professor University of Kent Ashford UK; ^2^ Restorative Dentistry and Periodontology Bristol Dental School University of Bristol and Secretary General European Federation of Periodontology Bristol UK; ^3^ Dentistry and Chair College of General Dentistry London UK; ^4^ ETEP Research Group Faculty of Odontology University Complutense of Madrid Madrid Spain

**Keywords:** 2020, Europe, periodontology, specialist training

## Abstract

**Aims:**

The survey's aim was to establish which universities and other educational organisations deliver postgraduate and specialist training in Periodontology in the 31 countries who are members of the European Federation of Periodontology (EFP) and to obtain details of how these programmes are organised, funded, regulated and evaluated.

**Methods:**

A questionnaire and covering letter were emailed to all national periodontal societies. The questions were on the name of country, official recognition, training programmes, entry to specialist training, specialist training assessment and recognition after completion of training.

**Results:**

Twenty‐nine (93%) of national periodontal societies responded. Key findings included the following: Periodontology was reported as being recognised at a national level in 17 countries, there was a three‐year full‐time programme in 12 countries, no fees were charged for specialist training in 10 countries, in 14 countries trainees received annual salaries, end of training (summative) assessments varied from country to country, 12 countries reported that they had a requirement for specialists in Periodontology to complete continuing education to maintain registration as specialists.

**Conclusions:**

This survey has established which universities and other educational organisations deliver postgraduate and specialist training in Periodontology and how these programmes are organised, funded, regulated and evaluated. To provide uniformly high‐quality periodontal care for patients in all European countries, further harmonisation of postgraduate and specialty training in Periodontology would be advantageous.

## 1 INTRODUCTION

Fifteen years have passed since the European Parliament, and the European Council approved a common European Directive on the Recognition for Professional Qualifications.^1^ Among other things, this directive sets out the educational requirements for the mutual recognition of certificates, diplomas and degrees in dentistry. Article 24 of the Directive defines "specialist dental training." Only two dental specialties (Orthodontics and Oral Surgery) are recognised by Directive 2005/36/EC.

Sanz et al. (2006) reported that Periodontology was recognised as a specialty at national level in 11 of the 25 European Union (EU) Member States.^2^ Subsequently, this number increased to 12 but reverted to 11 when the UK left the EU. All 11 Member States which have independently recognised Periodontology as a specialty also regulate the specialty, that is, only individuals recognised as specialists are entitled to use the title “specialist.” As such, Periodontology satisfies the key criteria for inclusion in the list of recognised EU specialities, which is that the specialty concerned is independently recognised and regulated in at least two‐fifths of EU Member States.^3^ The European Federation of Periodontology (EFP) has been lobbying for a number of years for general recognition of Periodontology as a specialty of dentistry by the European Commission.

In 1996, to promote standardisation of high‐quality postgraduate education in the specialty, the EFP published *Quality Standards for Graduate Programmes in Periodontology*. This document provided a basis for an EFP‐accredited Postgraduate Programme in Periodontology with a three‐year full‐time specialist training programme which follows a common, core curriculum.^4^ To date, this programme is offered by universities in 12 of the 31 countries whose national societies are full or associate members of the EFP. Two of the 31 countries are not EU member States and the UK is no longer. The regulations and structure of postgraduate and specialist training in Periodontology in the other 23 countries, whose periodontal societies or associations are members of the EFP, are currently unknown to the EFP.

Although there has been a study which investigated periodontal education and assessment in the undergraduate curriculum in European countries^5^ and an earlier study,^6^ which surveyed specialist orthodontic education in 23 European countries provides some relevant information, there have been no surveys which have investigated postgraduate and specialist training in Periodontology in Europe. Against this background, the Council of the EFP approved the survey reported in this paper.

## 2 AIMS

The aim of the survey was to establish which universities and other educational establishments deliver postgraduate and specialist training in Periodontology in the 31 countries whose national periodontal societies/associations are members of the EFP and to obtain details of how these programmes are run, funded, regulated and evaluated.

## 3 METHODS

In April 2020, the EFP office co‐ordinator emailed the presidents and secretaries of all 31 national periodontal societies/associations who are members of the EFP with a questionnaire and a covering letter from the EFP Secretary General. The questionnaire contained 28 questions, which were grouped under the headings:

- Name of country and name of specialty
- Official recognition
- Training programmes
- Entry to specialist training
- Specialist training
- Assessment
- Recognition after completion of training
- Additional comments

The questionnaire was based on the questionnaire used in a survey of orthodontic specialist training.^6^ In 2019, additional questions were added. The resulting version was piloted and then used in an internal survey of all dental specialties in Europe to advise Health Education England (HEE), as part of Phase II of the Advancing Dental Care Review.^7^ As such, it was decided that there was no need to pilot the questionnaire for a second time, prior to its distribution. All the questions in the questionnaire appear in the results section of this paper.

A covering letter explained the purpose of the survey and reassured respondents that they would not be named in any paper or presentation which reported the results of the survey. Respondents indicated their written consent to taking part in the survey by ticking a box at the top of the first page of the questionnaire.

Reminders were sent to non‐responders by email in May and June 2020. After an analysis of the responses, respondents were sent a copy of the results in June 2020 with a covering email asking them to check that the results for their country had been reported accurately.

The survey was approved by the EFP's Executive, who had consent from the national societies. In view of this and the facts that it was an internal survey and that respondents were not personally identified in publications arising from the survey, ethics approval was deemed not to be required.

## 4 RESULTS

### 4.1 Name of country and name of specialty

1. Responses from countries

Respondents from 29 countries out of a possible 31 completed or partially completed the questionnaire. They were:

Austria, Azerbaijan, Belgium, Croatia, Denmark, Finland, France, Georgia, Germany, Greece, Hungary, Ireland, Israel, Italy, Lithuania, Morocco, Netherlands, Norway, Poland, Portugal, Romania, Serbia, Slovenia, Spain, Sweden, Switzerland, Turkey, Ukraine and United Kingdom (UK). There were no responses from the Czech Republic and Russia.

2. Name of specialty

Of the 29 countries, respondents from 26 reported that the specialty was known as Periodontology or Periodontics. In Finland, it is a sub‐specialty of Clinical Dentistry. In Germany, periodontists may be described as either Fachzahnarzt fȕr Parodontologie or Spezialist/Spezialisten fȕr Parodontologie. In Serbia, at the time of the survey, the specialty was known as Periodontology and Oral Medicine.

### 4.2 Official recognition

3. Is Periodontology an officially recognised specialty in your country?

Respondents from all 29 countries answered this question. Seventeen replied yes and 11 no. In Germany, the specialty is recognised only in one Lande (Federal State), which is Nordrhein‐Westfalen.

4. Who keeps a list of officially recognised specialists in your country?

Respondents from 17 countries plus Germany reported that the organisation(s) keeping the list were as follows:

The Ministry of Health in nine countries,

The Dental Chamber (Association) and/or Periodontology Society in association with Ministry of Health in three countries,

The Dental Chamber (Association) and/or Periodontology Society in three countries, a Government Agency in two countries,

An Independent national regulator for dentistry in one country. 1

In the other 11 countries, it was reported that the specialty was not officially recognised but periodontal societies keep lists of those who have completed formal (three years) training in the specialty in two and that there was no list as the specialty was not recognised in the other nine countries.

### 4.3 Training programmes

5. Are there Masters or other postgraduate training programmes in dental specialties (such as Periodontology), in your country, which do not lead to official recognition as a specialist?

Respondents from 17 countries replied “yes” and gave details in answer to question 6.

Eleven countries replied “no.” There was no reply to this question from one country.

6. If “yes,” please list the Masters programmes.

Respondents reported that 52 universities in the 17 countries ran Masters or Masters/Doctoral programmes in periodontology. Sixteen universities in 12 of these countries ran the three‐year EFP‐approved Periodontal course.

### 4.4 Entry to specialist training

7. Is a period of postgraduate training and/or experience in general dentistry required before starting training in your specialty (Periodontology)?

8. If “yes,” how many years since initial qualification as a dentist?

Respondents from 24 countries described the entry process to specialist training in their country. Twelve countries require a period of postgraduate training and/or experience in general dentistry before starting training in periodontology, and this is not required in the other 12. Where the requirements are present, they range from one to three years, with two years the most frequently reported figure.

### 4.5 Specialist training

9. How many years full‐time does specialist training in your specialty (Periodontology) take?

Respondents from 22 countries answered this question. In twenty‐one countries, the answer was answered three‐year full‐time and in one, three and a half years full‐time.

10. Can specialist training in Periodontology be completed part‐time?

Respondents from 23 countries responded to this question with the answer no from 16 countries and yes from 7.

11. Does it take place only in a university?

Respondents from 23 countries responded to this question with the answer yes from 14 countries and no from nine.

12. If specialist training takes place in additional locations (outside a university), please list them and state what percentage of time takes place at these locations.

Respondents from nine countries reported that specialist training takes place outside a university for at least some time during the training programme. The locations were in: hospitals, approved public clinics, approved private periodontal practices and in one country in military clinics.

The percentage of time spent in these locations varied from 10% to 80% of the three‐year full‐time course.

13. Does a specialist trainee have to pay for their specialist training?

Respondents from 27 countries answered this question with yes in 13 countries, no in a further 13 countries and no for 3‐year programmes but yes for 1 year Masters programmes in one country.

14. If they have to, how much do they pay per year?

In the countries, where trainees were reported as having to pay for their training, the annual fees ranged from €1400 to €47 000 for trainees from non‐EU countries.

15. During training do trainees receive a salary?

Respondents from 23 countries responded to this question with the answer yes from 14 countries and no from nine.

16. If “yes” who pays the salary to university/hospital/government/other organisation?

Of the 14 countries which reported that trainees were paid, only two countries answered university only. In others, payment was usually from more than one source which frequently included a hospital in combination with a university or the government or the Health Ministry.

17. If other organisations (pay trainees) which one(s)?

Apart from the organisations mentioned in the answers to question 16, payments were reported as coming from public clinics in one country and private dental practices/offices in three. Respondents from two countries reported that, if trainees are in the military, they are paid salaries by the military whilst they train.

18. How much salary per year are trainees paid?

Reported annual salaries ranged from €2500 to €47 500.

### 4.6 Assessment

19. Are trainees assessed during training?

Respondents from four countries did not answer this question. In all the other 25 countries, it was reported that trainees were assessed during training.

20. If “yes,” who is responsible for assessment examinations?

In 24 of the 25 countries, it was reported that assessment was by a university, in the following countries in collaboration with other organisations as follows:

With hospitals in four countries.

Within practice by mentors or specialist supervisors in three countries.

With Royal Colleges of Surgeons in one country.

21. Is there an end of training examination?

“Yes,” in all 25 countries who responded to question 20.

22. If “yes,” who is responsible for the end of training assessment examination?

Respondents from 25 countries reported:

Solely universities in 11 countries.

Universities and government bodies in two countries.

Universities and hospitals in two countries, followed by approval from the National Board for Health and Welfare.

Universities and the EFP in three countries.

Universities and the specialist society in two counties.

Universities and Royal Colleges of Surgeons in one country.

National Examination Boards in three countries.

Ministry of Health in one country.

23. Which of the following aspects are included in the end of training assessment:

Written examination, oral examination, objective standardise examination (OSCE), presentation of a portfolio of treated cases, other (please specify).

Respondents from 25 countries reported that the following elements were included in the end of training assessment:

A written examination in 15 countries.

An oral examination in 20 countries.

An OSCE in 9 countries.

Presentation of a portfolio of treated cases in 22 countries.

A draft or published paper in one country.

A report of research in three countries.

Examination of previously unseen patients in one country.

### 4.7 Registration after completion of specialist training

24. Can those who have passed the end of training assessment register and work as specialists immediately?

Respondents from 15 of the 17 countries which were reported as having specialist lists for Periodontology answered that those who have completed an end of training assessment have no delay in registering and can work as specialists immediately.

In one country, it was reported that there is often a short wait, for administrative reasons, between completing specialist training and obtaining confirmation from the national registration body.

In the seventeenth country, those who have completed a three‐year full‐time training in Periodontology also have to pass an examination set by a Royal Colleges of Surgeons before they can apply to register as specialists.

25. If there is a wait between successfully completing specialist training and being able to work as Periodontal specialists, how long is it?

Respondents from only two countries reported that there was a wait. In one, there is no official specialist list for Periodontology. However, the Consilium Parodontologicum maintains a list of those who have successfully completed a three‐year full‐time training in Periodontology and during the two years after completion of training have built up a portfolio of treated cases and been successfully evaluated by the Consilium, which is run by the national Society of Periodontology, which then recognises them as periodontists.

On the other after successfully completing training in Periodontology, it is possible to work immediately as a specialist periodontist in the Public Dental Service. However, a further five years is required before newly qualified periodontists can work in private clinics as periodontists.

26. Are there any requirements in your country for those on a specialist register to be reassessed at regular intervals or to complete a minimum number of hours of continuing education in the specialty?

Respondents from 22 countries answered this question. From 13, the answer was yes, and from nine, it was no.

In two countries, specialists must renew their registration every 6 years by providing evidence of attending at least five periodontal congresses or seminars in Periodontology over the previous 6 years.

In ten, there is a requirement to attend approved oral health continuing education regularly and prove this to registering organisations at five yearly (seven yearly in two countries) intervals. The requirement in terms of /points credits/hours varies from country to country and it appears that in none of the countries, is it less than 100 h every five years or 140 h every 7 years.

In one, where the specialty is not officially recognised, the Periodontal society maintains a list of periodontal specialists and requires dentists to complete a wide range of tasks including completing at least 180 h of continuing dental education every five years and treating referred patients on at least 2 days per week.

27. In your country are those registered as dental specialists permitted to continue to work as general dentists?

There were responses to this question from 25 countries with the answer yes from 23 countries and no from two.

### 4.8 Additional comments

28. Additional Comments

Respondents from seven countries provided additional comments, which together with the full results can be viewed at www.efp.org.

## 5 DISCUSSION

Overall, the findings of the present survey indicated that there was considerable variation between European counties in postgraduate training in Periodontology, the mechanisms for funding such training, its assessment and the official recognition of the specialty. This mirrors the situation reported 20 years ago for orthodontic postgraduate and specialist training in Europe.^6^

With any survey, there may be doubts that the respondents fully understand the questions or interpret them differently. In order to address this potential problem, the questionnaire which was selected had been piloted and used in a recent survey of dental specialist training in Europe^7^ and the full set of results were sent to all respondents to ask them to check them and confirm that they were correct. The response rate of over 90% (29 countries out of 31) was considered to be excellent, with the findings therefore providing an clear picture of existing arrangements. The first inter‐country variation was in the name of the specialty. Although in 26 of the 29 countries, it was Periodontology or Periodontics, in three the specialty was included in, or grouped with other specialties. For example, currently, in Serbia, the specialty is Periodontology and Oral Medicine. However, the respondent from Serbia explained that the two specialties are likely to be recognised as individual specialties in the near future.

On the topic of recognition, the responses indicated that Periodontology was recognised nationally as a specialty in 17 EFP member countries, not all of whom are EU Member States. Essentially, three types of organisation, referred to in EU law as competent authorities,^1^ were reported as keeping lists of recognised specialists in Periodontology. In some countries, the competent authority was the Ministry of Health or, in Finland and Sweden, a "satellite" government agency. In others, it was the Dental Chamber (Dental Association), and in the UK, an independent, regulatory body (the General Dental Council). In some countries, the Society of Periodontology also maintained lists of periodontists. Within the EU, independent national recognition and regulation in at least two‐fifths of Member States are important for Periodontology to satisfy the key criterion for eligibility for specialty recognition by the European Commission.^3^ The EFP have been campaigning for this to happen for some years, and although eligibility has been confirmed, the goal has not yet been achieved, despite the many different ways in which the EU and its population would benefit from this development in professional freedom of movement.

As stated earlier, the EFP‐accredited three‐year full‐time postgraduate programme in Periodontology has been run for over 15 years by some dental schools. It is disappointing, according to the responses to the current survey, that it is still offered by only 16 dental schools in twelve countries. The question arises as to why dental schools which provide three‐year full‐time or equivalent part‐time courses have not sought EFP accreditation. It would be interesting also to establish if other dental schools which run Masters or other postgraduate courses are contemplating extending these programmes to three years and applying for EFP recognition. Such developments would be to the mutual advantage of forward‐looking universities and the EFP. One of the pillars of the European Federation of Periodontology's Strategic Plan is to encourage and promote education and training in Periodontology. The Federation needs to redouble its efforts to encourage universities to work towards their postgraduate programme in Periodontology being awarded EFP recognition.

The responses to the questions on entry to specialist training indicated that equal numbers of countries required or did not require experience in providing general dentistry prior to starting specialist training in Periodontology. In eight of the countries, which did require such experience, two years or more was required. As specialists in Periodontology, as in other specialties, require to understand the constraints under which many general dentists work or the general dentists' ability to manage clinical problems, it was disappointing to see that experience of general dental practice, prior to entering specialist training, was reported as not being required in 12 countries. Again, this begs the question of why, especially when many aspects of general dentistry underpin the provision of specialist services.

In 16 of the countries, specialist training in Periodontology had to be undertaken through a three‐year full‐time programme. However, in a further seven counties it could be completed by means of equivalent part‐time training. If trainees must pay for their training, this option makes it easier for trainees to fund as they have the opportunity to maintain an income on the days that they are not undertaking specialist training—“earn and learn.” Also, part‐time training provides the opportunity to remain in practice and acquire experience which typically compliments the experience gained in training. It was reported that no fees were charged for specialist training in 10 countries, and in a further 14, where fees were charged, the annual cost ranged from €1400 to €30 000 for trainees from EU Members States and up to € 47 000 for trainees from countries outside the EU. Trainees were paid during their training in 14 countries, and their annual salary ranged from €2500 to €45 000. The pay came from universities, hospitals, health ministries, and in one country, the specialist practice where they worked. Remuneration of specialty trainees is believed to be linked to local arrangements for funding specialist services.

The location of training varied. Generally, at least some if not all training took place in a university department, exclusively so in 14 countries. Nine countries reported that it also took place in hospitals and public and private clinics under the supervision of one or more periodontal specialists. In one country, if the trainees were military dentists, up to 14% of training could take place in army clinics and hospitals. Thus, wide variations were reported, in both the funding and location of periodontal training in the 29 countries who took part in the survey, possibly reflecting wide variations in the arrangements for the provision of specialist services across Europe. In 2009, during a workshop organised by the EFP and the Association for Dental Education in Europe (ADEE), it was concluded that the location where specialist education in Periodontology took place was irrelevant as long as the location had been accredited as fulfilling the competences and quality standards defined by the ADEE and EFP.^8^, ^9^

Unsurprisingly, in all 25 countries, which answered the relevant questions, assessment of trainees was reported as taking place both during and at the end of specialist training. The interim (formative) assessments were performed by universities, either solely or in combination with hospitals, specialist periodontists working in specialist clinics and, in the UK, Royal Colleges of Surgeons. End of training (summative) assessment was reported as being performed solely by universities in 11 countries, by national examination boards in three countries and by the Ministry of Health in one country. In other countries, end of training assessment was performed by universities in combination with a number of other organisations, which varied from country to country and were as follows: government bodies, hospitals, the EFP, specialist societies and Royal Colleges of Surgeons. In the 25 countries, which answered the relevant question, 23 included an oral examination and 22 the presentation of a portfolio of treated cases in the summative assessment. Sixteen also included a written examination and nine Objective Standardised Clinical Examinations (OSCEs), three required a thesis or the publication of a paper in a peer‐reviewed journal and one diagnosis and treatment planning for "unseen" cases. In all 25 countries, more than one method was used for summative assessment. A previous study found even greater differences in end of training assessment for Orthodontic specialty training.^6^ Although the EFP has endeavoured to standardise the summative assessment of periodontal specialty trainees across Europe, specifically in relation to competences, the results of the current survey indicate that further work is required. To date, there is no evidence of any significant differences in the competences of newly qualified specialty trainees in Periodontology across Europe; however, confirmation of this assertion would be advantageous.

Answers to the questions on the topics of registration after successful completion of training and commencing work as a periodontal specialist indicated that there was usually minimal delay in registering as a specialist with the relevant national body (competent authority), in countries where the specialty is recognised nationally. However, in two countries there were some limitations on the freedom to practice immediately as a specialist in Periodontology. In the Netherlands, where Periodontology is not officially recognised as a specialty, as reported in the results section of this paper, those who have successfully completed a three‐year postgraduate programme in Periodontology have to build up a portfolio of treated cases and have them successfully evaluated by the Dutch Society of Periodontology before this Society recognises them as periodontists. In Slovenia, individuals who successfully complete specialty training in Periodontology can only work as specialists in Periodontology in the public dental service and must wait five years before they can practice as specialists in private clinics. In two countries (Belgium and France), dental specialists (including periodontists) are not permitted to work as general dentists. This restriction can and does cause inconvenience to patients and considerably limits the scope of the dentists concerned. Such arrangements may be difficult to sustain with the international shift to the integrated, holistic care of patients, with high‐quality general dentistry underpinning the provision of specialist services.

As far as a requirement for trained periodontists to complete a minimum number of hours of continuing education (CE) in Periodontology was concerned, this was found to be obligatory in 12 countries. In most of these countries, the requirement was for at least 100 hours of CE within a five‐year period. However, in the Netherlands, the Dutch Society of Periodontology has stringent requirements, for individuals to remain on the Society's list of specialists. Other countries have continuing professional development requirements to ensure high‐quality standards are maintained.

Two previous studies have identified the need for common standards in postgraduate and specialist training in Periodontology in Europe.^2^, ^10^ It is therefore disappointing that the EFP‐accredited Postgraduate Programme is available in only a minority of European countries whose national periodontal societies are members of the EFP. Harmonisation of at least core specialty training across Europe would have greatly aided the EFP’s campaign for EU specialty recognition, let alone reinforce the assertion that specialists in Periodontology across Europe share the same competences.

## 6 CONCLUSIONS

This survey has established which universities and other organisations carry out postgraduate and specialist training in Periodontology in the 31 countries whose national periodontal societies/associations are full or associate members of the EFP Details of how these programmes are organised, funded, regulated and evaluated have been reported. To provide uniformly high‐quality periodontal care for patients in all European countries, further harmonisation of postgraduate and specialty training in Periodontology would be advantageous.

## CONFLICT OF INTEREST

The authors do not have any conflicts of interests.

## AUTHOR CONTRIBUTIONS

KAE designed the survey, drafted the covering letter, analysed the results, drafted the paper and approved its final version. NXC obtained approval for the survey, reviewed the progress of the project at all stages, revised drafts and approved the final version of the paper. NHFW contributed to the preparation of the report and approved the final version of the paper. MS contributed to the preparation of the report and approved the final version of the paper.

## Data Availability

The data that support the findings of this study are available from the corresponding author upon reasonable request, and a more detailed results section of the report of the survey can be accessed from the Publications and Education section of the EFP's website www.efp.org.

## References

[1] European Commission Directive 2005/36/EC. Official Journal of European Communities 2005, L255:0022‐0142.

[2] Sanz M , Van der Velden U , van Steenberghe S , Baehni P . Periodontology as a recognised dental specialty in Europe. J Clin Perio. 2006;33:371‐375. 10.1111/j.1600-051X.2006.00932.x 16677324

[3] European Commission Directive 2013/55/EU (amending Directive 2005/36/EC). Official Journal of the European Union. 2013. L354/132.

[4] European Federation of Periodontology . EFP accredited Postgraduate Programme in Periodontology 2017. https://www.efp.org/education/postgraduate/index.html. Accessed 28 September 2020.

[5] Gȕrsoy M , Wilensky A , Claffey N , et al. Periodontal education and assessment in the undergraduate dental curriculum‐A questionnaire‐based survey in European countries. Eur J Dent Ed. 2018;22(3):e488‐e499. 10.1111/eje.12330 29460375

[6] McDonald JP , Adamidis JP , Eaton KA , Seeholzer H , Sieminska‐Piekarczyk BA . Survey of postgraduate (specialist) orthodontic education in 23 European countries. J Ortho. 2000;27:92‐98. 10.1093/ortho/27.1.92 10790453

[7] Health Education England . Advancing dental care review. Advancing Dental Care Phase II. 2019. https://www.hee.nhs.uk/our‐work/advancing‐dental‐care. Accessed 29 September 2020.

[8] Sanz M , Meyle J . Scope, competences, learning outcomes and methods of periodontal education within the undergraduate dental curriculum: A Consensus report of the 1st European workshop on periodontal education ‐ position paper 2 and consensus view 2. Eur J Dent Ed. 2010;3:25‐33. 10.1111/j.1600-0579.2010.00621.x 20415973

[9] der Velden UV , Sanz M . Postgraduate periodontal education. Scope, competences, proficiencies and learning outcomes: Consensus report of the 1st European workshop on periodontal education. Eur J Dent Ed. 2010;14(SUPPL. 1):34‐40. 10.1111/j.1600-0579.2010.00622.x 20415974

[10] Sanz M , Widström E , Eaton KA . Is there a need for a common framework of dental specialties in Europe. Eur J Dent Ed. 2008;12:138‐143. 10.1111/j.1600-0579.2008.00510.x 18666894

